# Anti-Rubella antibodies seroprevalence among pregnant women in South-Western Romania

**DOI:** 10.3389/fmed.2026.1906798

**Published:** 2026-09-24

**Authors:** Luiza Cristiana Radoi-Troaca, Oana Mariana Cristea, Ovidiu Mircea Zlatian, Maria Balasoiu, Alice Elena Ghenea, Lidia Boldeanu, Dominic Gabriel Iliescu

**Affiliations:** 1 Department of Infections Diseases, University of Medicine and Pharmacy of Craiova, Craiova, Romania; 2 Medical Laboratory, County Clinical Emergency Hospital of Craiova, Craiova, Romania; 3 Department of Microbiology, University of Medicine and Pharmacy of Craiova, Craiova, Romania; 4 Department of Obstetric and Gynecology, University of Medicine and Pharmacy of Craiova, Craiova, Romania; 5 Obstetric and Gynecology Department, County Clinical Emergency Hospital of Craiova, Craiova, Romania

**Keywords:** antibodies, congenital infections, pregnant women, rubella, seroprevalence studies

## Abstract

**Introduction:**

Rubella infection during pregnancy poses significant risks, including congenital rubella syndrome (CRS), miscarriage, and fetal death. Vaccination programs have reduced rubella incidence globally, but regional differences in coverage and immunity persist. The objective of the study was to assess the seroprevalence of anti-rubella IgM and IgG antibodies among pregnant women in South-Western Romania, with a focus on temporal trends, demographic factors, and the impact of vaccination eligibility.

**Methods:**

This observational, cross-sectional study included pregnant women screened for TORCH infections at the County Clinical Emergency Hospital of Craiova, Romania, during two periods: 2013–2016 and 2019–2022. Serum samples were tested for anti-rubella IgM and IgG antibodies. Participants were stratified by age, area of residence (rural/urban), and eligibility for rubella vaccination based on birth year.

**Results:**

A total of 3,268 samples were analyzed. The seroprevalence of acute rubella infection (IgM positive) was low and slightly decreased from 0.54% (2013–2016) to 0.50% (2019–2022), with the most notable decrease in rural areas and among women aged 21–25 years. The overall seroprevalence of protective IgG antibodies was stable (88.28% vs. 88.91%), but remained below the WHO target of 95%. IgG seroprevalence increased among the youngest (<20 years) and 31–35 years age groups, particularly in rural areas, suggesting improved vaccination coverage and natural immunity from past outbreaks. Mapping in Dolj County revealed high seroprevalence near major cities.

**Discussion:**

Despite improvements, the relatively low IgG seroprevalence in South-Western Romania highlights the need to reinforce vaccination campaigns, especially in rural areas and among family physicians. Enhanced education and access to vaccination are essential to achieve the WHO eradication target and prevent CRS.

## 1 Introduction

Rubella is an airborne viral infection caused by the rubella virus, a Rubivirus in the family Matonaviridae (1) (reclassified from the former family Togaviridae following the 2018 ICTV taxonomy revision). Following the widespread introduction of rubella-containing vaccines (RCVs), global rubella incidence declined substantially, from over 670,000 reported cases in 2000 to fewer than 15,000 cases in 2018 (2). Nevertheless, rubella has not been eliminated, and sporadic cases continue to be reported in Europe, particularly in countries with suboptimal vaccination coverage (3).

Although rubella is usually a mild, self-limiting disease in children and non-pregnant adults, maternal infection during pregnancy can result in severe adverse outcomes, including miscarriage, stillbirth, fetal death, and congenital rubella syndrome (CRS) (4, 5). The risk of CRS is strongly dependent on gestational age at infection, being highest during the first trimester and decreasing thereafter (5). For this reason, rubella immunity among women of childbearing age remains a critical public health concern, even in the post-vaccination era.

CRS is a preventable condition characterized by permanent disabilities, most commonly sensorineural deafness, cardiac malformations, ocular abnormalities, and neurodevelopmental impairment (2, 5). The introduction of RCVs has dramatically reduced the global burden of CRS; however, immunity gaps persist where vaccine coverage is incomplete or heterogeneous. These gaps may remain undetected until susceptible women reach reproductive age, at which point the consequences of infection become clinically and socially significant.

The World Health Organization (WHO) has set rubella and CRS elimination targets that require sustained high vaccination coverage, ideally exceeding 95% with rubella-containing vaccines (6). Despite progress, global RCV coverage was estimated at only 68% in 2022, and marked differences persist between regions and countries (7). In Europe, although two doses of the measles–mumps–rubella (MMR) vaccine are included in national immunization schedules, vaccination coverage remains uneven. Countries such as Romania and Poland continue to report lower coverage rates compared with Western European states (3).

In Romania, rubella vaccination was introduced relatively late (Figure 1). A monovalent rubella-containing vaccine was administered to adolescent girls between 2003 and 2008, followed by the inclusion of the MMR vaccine in the childhood immunization program starting in 2004 (7, 8). As a result, several birth cohorts experienced incomplete or inconsistent vaccination exposure, creating potential immunity gaps that may persist into adulthood. Previous outbreaks in Romania, most notably in 2003 and 2011–2012, disproportionately affected these cohorts and highlighted the vulnerability of unprotected women of reproductive age.

**Figure 1:**
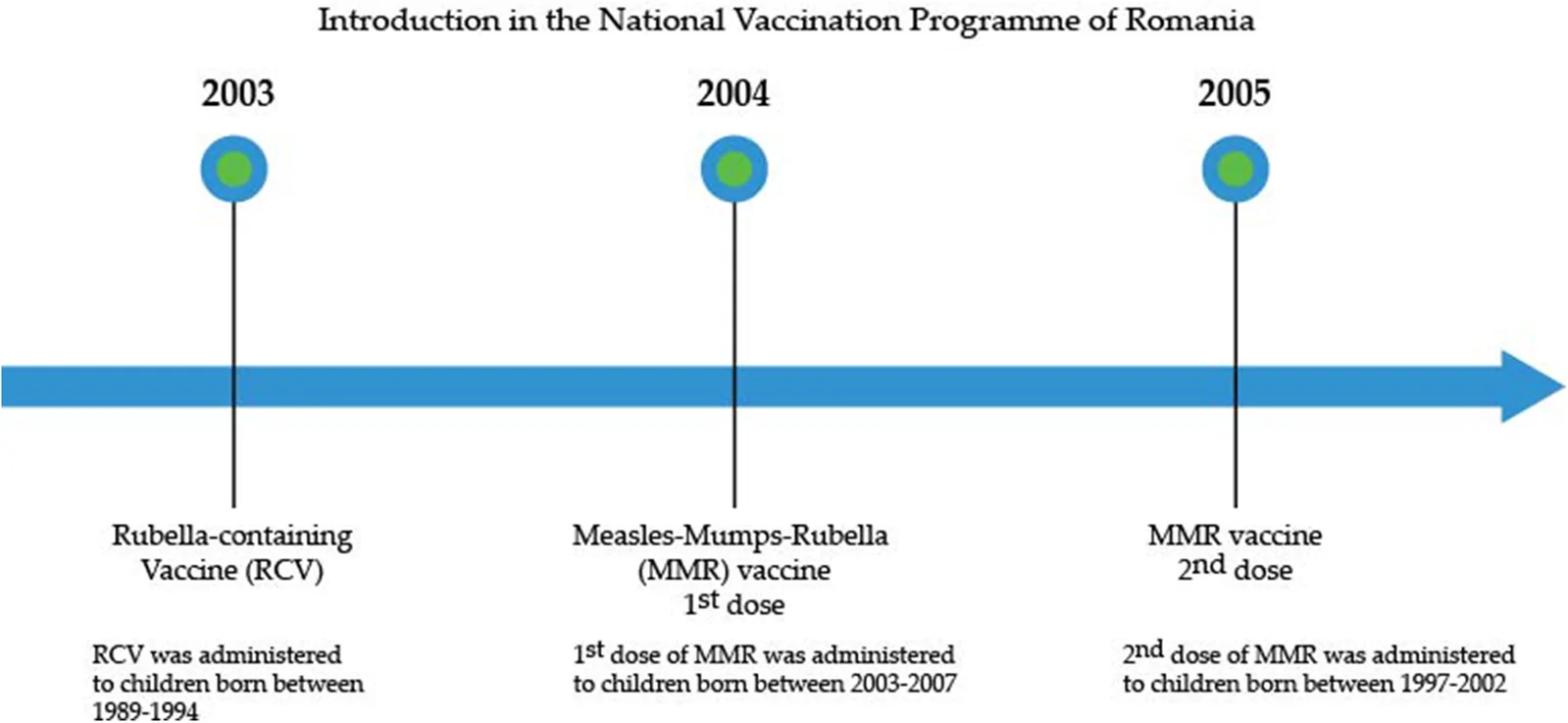
History of vaccination anti-Rubella in Romania.

Pregnant women represent a sentinel population for assessing population-level rubella immunity, as insufficient protection directly translates into the risk of CRS. However, contemporary seroprevalence data from South-Western Romania remain limited. The present study aimed to assess the seroprevalence of anti-rubella IgG and IgM antibodies among pregnant women over two distinct periods (2013–2016 and 2019–2022) and to identify immunity gaps according to age, area of residence, and vaccination eligibility cohorts.

## 2 Material and methods

### 2.1 Study design and participants

This observational, cross-sectional study included pregnant women from South-Western Romania who presented for routine TORCH screening at the Gynecology Department of the County Clinical Emergency Hospital of Craiova, Romania. Two study periods were analyzed (2013–2016 and 2019–2022). In the first period, 368 women were tested for IgM and 367 for IgG, compared with 2,779 and 2,669 women, respectively, in the second period. Not all participants were tested for both antibodies: some women were tested only for IgM or only for IgG as part of routine clinical care, which explains the difference between the number of women tested for each antibody; no additional exclusion criteria were applied beyond having at least one valid rubella serology result.

### 2.2 Sample size calculation

According to the National Institute of Statistics, the South-Western (Oltenia) region of Romania had 974,202 women of childbearing age (15–49 years) in 2019 (9). Using the lowest regional rubella IgG seroprevalence reported in Romania at the time of study design (91.4%) (10), we estimated that a minimum sample of 121 pregnant women would be required to estimate seroprevalence with a two-sided 95% confidence interval and a ± 5% margin of error, using the standard formula *n* = Z^2^p(1−p)/d^2^ (Z = 1.96). The achieved sample sizes for IgG testing (367 in Group 1 and 2,669 in Group 2) exceeded this minimum, corresponding to margins of error of approximately ±2.87% and ±1.06%, respectively; the smaller Group 1 sample therefore yields comparatively less precise estimates, a point considered further in the Limitations.

### 2.3 Data collection and variables

For each participant, demographic data including age and area of residence (urban or rural) were recorded, together with the results of anti-rubella IgM and IgG serological testing.

### 2.4 Laboratory testing

Pregnant women were screened during the first trimester of pregnancy as part of routine prenatal care.

Between 2013 and 2016, anti-rubella IgM and IgG antibodies were measured using an electrochemiluminescence immunoassay (ECLIA) on the Cobas e601 analyzer (Roche Diagnostics, Basel, Switzerland). Between 2019 and 2022, serological testing was performed using a chemiluminescent microparticle immunoassay on the Architect i1000 system (Abbott Laboratories, Abbott Park, IL, USA) with original reagents. Both platforms report anti-rubella IgG results in international units per milliliter, calibrated against the WHO International Standard for anti-rubella immunoglobulin. However, multicentric evaluations of commercial rubella IgG immunoassays, including these two platforms, have shown that quantitative titers and, to a lesser extent, qualitative classification near the assay cut-off may differ between platforms despite this common calibration (11). This potential source of inter-assay variability should be considered when interpreting between-period comparisons and is discussed further in the Limitations.

### 2.5 Statistical analysis

Data were exported from the hospital electronic database (HIPOCRATE) and analyzed using STATA version 17 (StataCorp LLC, College Station, Texas, USA). Continuous variables were expressed as mean ± standard deviation, while categorical variables were reported as counts and percentages.

Comparisons between the two study periods were performed using the chi-square test for categorical variables. Differences in mean age between groups were assessed using the Student's *t*-test, as age was normally distributed. A *p*-value <0.05 was considered statistically significant.

Because this is a cross-sectional study, associations between vaccination-eligibility cohorts (or area of residence) and antibody seroprevalence are expressed as prevalence ratios (PR) with 95% confidence intervals (CI), rather than risk ratios, in line with recommended practice for prevalence (rather than incidence or risk) data (12). PR estimates were calculated as the ratio of the crude prevalence in each comparison subgroup to that of the reference subgroup (women born before 1989, not eligible for rubella vaccination), with 95% CIs derived using the standard log-linear approximation and a continuity correction (+0.5) applied to comparisons involving zero-count cells.

IgM seropositivity was interpreted descriptively as a possible marker of recent or acute rubella infection and was not used to estimate population-level rubella immunity; a positive IgM result alone does not equate to confirmed acute infection, since definitive confirmation would require IgG avidity testing or reverse-transcription PCR, both of which were outside the scope of this cross-sectional serological survey.

### 2.6 Vaccination eligibility cohorts

Participants were stratified into four subgroups based on year of birth as a proxy for eligibility for rubella vaccination in Romania, in accordance with the national immunization schedule (Table 1).

**Table 1:** Correspondence between birth year and eligibility for rubella vaccine in Romania.

Birth year	<1989	1989	1990	1991	1992	1993	1994	1995	1996	1997	1998	1999	2000	2001	2002	2003	2004	2005	2006	2007
Age group G1	26–30	21–25	21–25	21–25	21–25	<20	<20	<20	<20	<20	<20	–	–	–	–	–	–	–	–	–
Age group G2	31–35	26–30	26–30	26–30	26–30	26–30	21–25	21–25	21–25	21–25	21–25	<20	<20	<20	<20	<20	<20	–	–	–
Eligible RCV	NO	YES D1	YES D1	YES D1	YES D1	YES D1	YES D1	–	–	–	–	–	–	–	–	–	–	–	–	–
Eligible MMR	NO	NO	NO	NO	NO	NO	NO	NO	NO	YES D2	YES D2	YES D2	YES D2	YES D2	YES D2	YES D1 + 2	YES D1 + 2	YES D1 + 2	YES D1 + 2	YES D1 + 2

D1, one dose of RCV vaccine.

D2, second dose of MMR vaccine.

D1 + 2: both doses of MMR vaccine.

Women born before 1989 were considered non-eligible for rubella vaccination, as they were older than 15 years at the time of introduction of the rubella-containing vaccine in 2003. Women born between 1989 and 1994 were eligible for the monovalent rubella-containing vaccine (RCV) administered to adolescent girls between 2003 and 2008. Women born between 1995 and 1996 constituted a vaccination gap cohort, as they were not eligible for either the RCV program or the childhood MMR schedule. Women born in 1997 or later were considered eligible for the MMR vaccine; those born between 1997 and 2002 received only the second MMR dose, while those born from 2003 onward were eligible for both doses.

Women born between 1997 and 2002, listed in Table 1 as eligible only for the second MMR dose, received a first dose of monovalent measles vaccine (without a rubella component) in infancy, before the MMR vaccine replaced it in the national immunization schedule in 2004; their first exposure to a rubella-containing vaccine therefore occurred with the second MMR dose, administered at 6–7 years of age, typically as a school-based catch-up dose (7, 10, 13). This is also the only birth cohort in this classification, together with the 1989–1994 RCV-eligible cohort, that received a rubella-containing vaccine as a single dose rather than the two-dose MMR schedule.

### 2.7 Institutional review board statement

The study was conducted according to the ethical principles regarding human experimentation and clinical research, and was approved by the Committee of Ethics and Academic and Scientific Deontology, Craiova, Romania (approval no. 84/16.09.2020).

## 3 Results

We tested in total 3,268 samples for anti-Rubella antibodies (388 samples from Group 1 and 2,880 samples from Group 2. In Group 1, we tested 368 samples for IgM and 367 samples for IgG, whereas in Group 2, we tested 2,779 samples for IgM and 2.669 samples for IgG (Figure 2).

**Figure 2:**
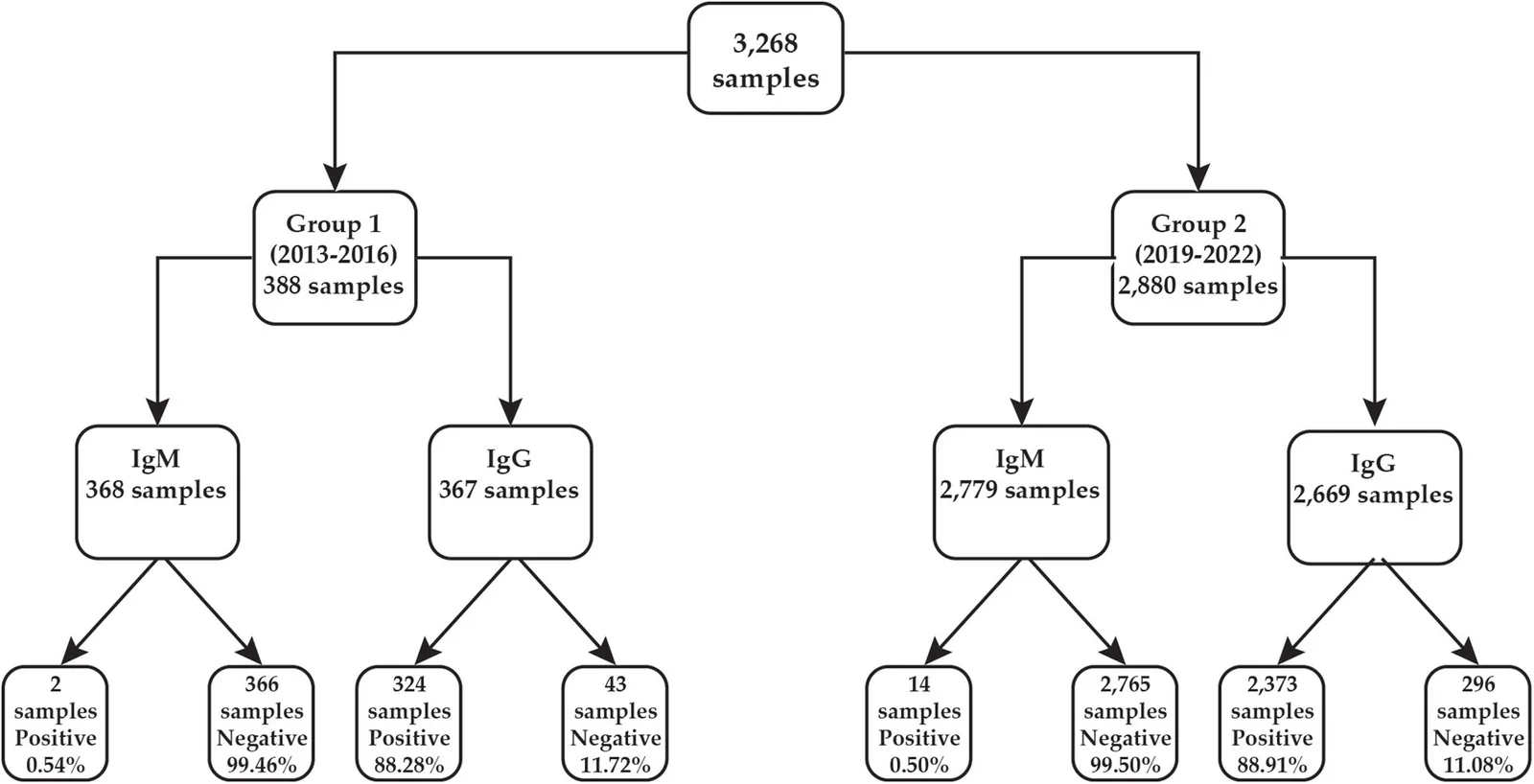
The results of testing the anti-Rubella IgM and IgG antibodies.

### 3.1 Acute Rubella infection (anti-Rubella IgM antibodies)

Anti-rubella IgM testing included 368 women in Group 1 (2013–2016) and 2,779 women in Group 2 (2019–2022). IgM positivity was rare and stable over time (0.54% in Group 1 vs. 0.50% in Group 2), with no statistically significant differences after stratification by residence, age group, or vaccination-eligibility cohorts (supplementary Tables S1, S2).

We further stratified the seroprevalence in the four subgroups by eligibility for Rubella vaccine, based on the year of birth of the pregnant women (Table 1, Table 2).

**Table 2:** Anti-Rubella IgM positivity in pregnant women in 2013–2016 (group 1) versus 2019–2022 (group 2), overall and stratified by area of residence and age group.

	**GROUP 1 (201–2016)** ***n*** **=** **368**							
	<1989 *n* = 184		1989–1994 *n* = 124		1995–1996 *n* = 25		>=1997 *n* = 35	
Stratum	No (%)	PR^1^ (95% CI)	No (%)	PR^1^ (95% CI)	No (%)	PR^1^ (95% CI)	No (%)	PR^1^ (95% CI)
Total	0/184 (0%)	Ref.	2/124 (1.61%)	NE	0/25 (0%)	NA	0/35 (0%)	NA
Urban	0/51 (0%)	Ref.	1/53 (1.88%)	NE	0/15 (0%)	NA	0/26 (0%)	NA
Rural	0/133 (0%)	Ref.	1/71 (1.41%)	NE	0/10 (0%)	NA	0/9 (0%)	NA
PR^2^ (95% CI)	NA			NA		NA		NA

	**GROUP 2 (2019–2022)** **n=2,779**							
	<1989 *n* = 666		1989–1994 *n* = 855		1995–1996 *n* = 280		>=1997 *n* = 978	
Stratum	No (%)	PR^1^	No (%)	PR^1^	No (%)	PR^1^	No (%)	PR^1^
Total	1/666 (0.15%)	Ref.	5/855 (0.58%)	3.89 (95% CI 0.46–33.26)	2/280 (0.71%)	4.76 (95% CI 0.43–52.25)	6/978 (0.61%)	4.09 (95% CI 0.49–33.86)
Urban	0/396 (0%)	Ref.	4/438 (0.91%)	NE	0/104 (0%)	NA	1/277 (0.36%)	NE
Rural	1/270 (0.37%)	Ref.	1/417 (0.24%)	0.65 (95% CI 0.04–10.31)	2/176 (1.13%)	3.07 (95% CI 0.28–33.58)	5/701 (0.71%)	1.93 (95% CI 0.23–16.41)
PR^2^ (95% CI)	NA	-	3.81 (95% CI 0.43–33.93)	NA	-	NA	-	0.51 (95% CI 0.06–4.31)

PR^1^, prevalence ratio relative to the group born before 1989, not eligible for anti-Rubella vaccine.

PR^2^, prevalence ratio in women from urban areas relative to women from rural areas, within the same eligibility subgroup.

Ref, reference level.

NA, not applicable (reference group had zero events); NE, not estimable (comparison group had zero events).

In Group 1, we only had 2/124 (1.61%) positive results (one from rural areas and one from urban areas) for IgM antibodies in the subgroup eligible for vaccination with the RCV vaccine (born between 1989 and 1994), whereas in the other subgroups, there were no positive results.

In Group 2, the highest seroprevalence was observed in women born between 1995 and 1996 [0.71%; Prevalence Ratio (PR) = 4.76, 95% CI 0.43–52.25, *p* = 0.208], while the lowest were in the women born before 1989 who were not eligible for anti-Rubella vaccination (0.15%). Given the very small number of IgM-positive cases in this subgroup (2/280), this prevalence ratio should be interpreted with caution, as reflected in its wide confidence interval.

In rural areas, the highest seroprevalence (1.13%, PR = 3.07, 95% CI 0.28–33.58, *p* = 0.365) was in women born between 1995 and 1996 (vaccination gap subgroup), whereas the lowest was in those born between 1989 and 1994 (eligible for RCV vaccine). We observed a higher seroprevalence (0.91%) in urban areas than in rural areas (0.24%) (PR = 3.81, 95% CI 0.43–33.93) in the subgroup born between 1989 and 1994, conversely, a lower seroprevalence was observed in urban areas (0.36%) and rural areas (0.71%) in the subgroup born in or after 1997 (PR = 0.51, 95% CI 0.06–4.31) eligible for the MMR vaccine (Table 2). Because of the very small number of events in most of these subgroups, all of these urban-rural comparisons have wide confidence intervals crossing 1 and should be regarded as exploratory rather than conclusive.

Because this is a cross-sectional study, associations are expressed as prevalence ratios (PR) with 95% confidence intervals (CI), rather than risk ratios, following recommended practice for prevalence data (14). PR and 95% CI were calculated directly from the tabulated counts using the standard log-linear approximation, with a continuity correction (+0.5) applied to cells containing zero counts. Differences between periods were assessed using chi-square/Fisher's exact tests as appropriate; *p* < 0.05 was considered statistically significant.

### 3.2 Rubella seroprevalence (anti-Rubella IgG antibodies)

#### 3.2.1 Demographic characteristics

In our study, in the first period (2013–2016) we tested 367 pregnant women for anti-Rubella IgG antibodies and 2,669 in the second period (2019–2022). Pregnant women from rural areas had a significantly lower proportion of 145/367 (39.51%) between 2013 and 2016 (Group 1) than between 2019 and 2022 (Group 2) (1,500/2,669, 56.20%, *p* < 0.001) (Table 3). Conversely, we observed a statistically significant decrease in the proportion of women tested in urban areas from Group 1 (223/367, 60.49%) to Group 2 (1,169/2,669, 43.80%; *p* < 0.001). We also observed that the average age of the subjects in Group 1 was almost 1 year older than that of those in Group 2 (*p* = 0.013) (Table 3).

**Table 3:** Demographic characteristics of the pregnant women tested for IgG anti-Rubella antibodies.

Characteristic	Group 1 *n* = 367	Group 2 *n* = 2,669	*p*
Area of residence (*n*/%)			
Rural	145/367 (39.51%)	1,500/2,669 (56.20%)	<0.001*
Urban	222/367 (60.49%)	1,169/2,669 (43.80%)	
Age (years)	28.42	27.58	0.013*

* Statistical significance (*p* < 0.05).

#### 3.2.2 Seroprevalence results

Comparing the total seroprevalence of anti-Rubella IgG antibodies between the two groups, we observed a similar value (88.28% in Group 1 vs. 88.91% in Group 2; PR = 1.01, 95% CI 0.97–1.05; *p* = 0.721), even though the number of subjects tested differed considerably between the two groups (*n* = 367 in Group 1 vs. *n* = 2,669 in Group 2). When we stratified by area of residence, seroprevalence was numerically similar between periods in both rural (86.21% vs. 88.20%; PR = 1.02, 95% CI 0.96–1.09; *p* = 0.480) and urban areas (89.64% vs. 89.82%; PR = 1.00, 95% CI 0.95–1.05; *p* = 0.935), with no statistically significant difference between periods in either setting (Table 4).

**Table 4:** Anti-rubella IgG seroprevalence in pregnant women in 2013–2016 (group 1) versus 2019–2022 (group 2), overall and stratified by area of residence and age group. *p*-values represent between-period comparisons within each stratum.

Variable	Group 1 2013–2016 (*n* = 367)	Group 2 2019–2022 (*n* = 2,669)	*p* value
Total prevalence	324/367 (88.28%)	2,373/2,669 (88.91%)	0.721
Area of residence			
Rural	125/145 (86.21%)	1,323/1500 (88.20%)	0.480
Urban	199/222 (89.64%)	1,050/1,169 (89.82%)	0.935
Age group			
< 20 years	22/28 (78.57%)	240/282 (85.11%)	0.362
21–25 years	71/79 (89.87%)	593/688 (86.19%)	0.363
26–30 years	117/128 (91.41%)	692/775 (89.29%)	0.467
31–35 years	66/80 (82.50%)	561/607 (92.42%)	0.003*
> 35 years	48/52 (92.31%)	287/307 (90.54%)	0.753
Area of residence and age group			
Rural, <20 years	18/22 (81.82%)	187/214 (87.38%)	0.462
Rural, 21–25 years	38/46 (82.61%)	407/476 (85.50%)	0.597
Rural, 26–30 years	39/44 (88.64%)	358/412 (86.89%)	0.743
Rural, 31–35 years	18/21 (85.71%)	255/267 (95.51%)	0.050*
Rural, >35 years	12/12 (100%)	116/131 (88.55%)	0.215
Urban, <20 years	4/6 (66.67%)	53/68 (77.94%)	0.529
Urban, 21–25 years	33/33 (100%)	186/212 (87.74%)	0.033*
Urban, 26–30 years	78/84 (92.86%)	334/363 (92.01%)	0.794
Urban, 31–35 years	48/59 (81.36%)	306/340 (90.00%)	0.050*
Urban, >35 years	36/40 (90.00%)	171/186 (91.94%)	0.367

* Statistical significance (*p* < 0.05). Because multiple subgroup comparisons are presented, statistically significant *p*-values should be interpreted cautiously.

Among the tested women, those age group 31–35 years showed a statistically significant increase in seroprevalence between the two time periods (82.50% vs. 92.42%, *p* = 0.003). We also observed an increase in the <20 years age group (78.57% vs. 85.11, *p* = 0.362). In the other age groups, seroprevalence decreased from 2013 to 2016 to 2019–2022. Stratifying by both variables, the increase in seroprevalence in the 31–35 years age group was statistically significant in both rural (85.71% vs. 95.51%, *p* = 0.050) and urban populations (81.36% vs. 90.00%, *p* = 0.050). We also observed a significant decrease in seroprevalence among women aged 21–25 years from urban areas (100% vs. 87.74%, *p* = 0.033) (Table 4).

We analyzed the seroprevalence of anti-rubella IgG antibodies in the four vaccination-eligibility subgroups, further stratified by area of residence, using prevalence ratios (PR) with 95% confidence intervals (CI) relative to the reference group born before 1989 (Table 5). In Group 1, IgG seroprevalence was significantly lower only in the subgroup born in or after 1997, both overall (24/34, 70.58% vs. 162/183, 88.52%; PR = 0.80, 95% CI 0.64–1.00) and in urban areas (6/9, 66.67%; PR = 0.76, 95% CI 0.48–1.21); the rural subgroup showed a reduction of similar magnitude (18/25, 72.00%; PR = 0.80, 95% CI 0.62–1.04). Seroprevalence in the subgroups eligible for the RCV vaccine (born 1989–1994) or born during the 1995–1996 vaccination-gap did not differ significantly from the reference group (Table 5). In Group 2, IgG seroprevalence was significantly lower in the subgroup born between 1995 and 1996, both overall (223/269, 82.89% vs. 581/645, 90.07%; PR = 0.92, 95% CI 0.87–0.98, *p* = 0.003) and in rural areas (138/173, 79.76%; PR = 0.89, 95% CI 0.82–0.97), as well as in the subgroup born in or after 1997 (799/923, 86.56%; PR = 0.96, 95% CI 0.93–1.00). In this same group, seroprevalence in the subgroup eligible for the RCV vaccine (born 1989–1994) was similar to, and slightly higher than, the reference group (773/832, 92.90%; PR = 1.03, 95% CI 1.00–1.06). Prevalence ratios comparing urban to rural areas within each eligibility subgroup were close to unity in both groups (PR2 = 0.97, 1.15, 1.14, and 0.93 for the <1989, 1989–1994, 1995–1996, and >=1997 birth cohorts in Group 1, respectively, and 1.02, 1.01, 1.11, and 0.97 in Group 2), indicating no consistent urban-rural disparity once the vaccination-eligibility cohort was accounted for (Table 5).

**Table 5:** Distribution of the seroprevalence of anti-Rubella IgG antibodies in pregnant women according to eligibility for Rubella vaccine.

	GROUP 1 (2013–2016) n = 367							
	<1989 *n* = 183		1989–1994 *n* = 124		1995–1996 *n* = 26		>=1997 *n* = 34	
Stratum	No (%)	PR^1^	No (%)	PR^1^	No (%)	PR^1^	No (%)	PR^1^
Total	162/183 (88.52%)	Ref.	112/124 (90.32%)	1.02 (95% CI 0.94–1.10)	24/26 (92.30%)	1.04 (95% CI 0.92–1.18)	24/34 (70.58%)	0.80 (95% CI 0.64–1.00)
Urban	116/132 (87.87%)	Ref.	68/71 (95.77%)	1.09 (95% CI 1.01–1.18)	10/10 (100%)	1.14 (95% CI 1.07–1.21)	6/9 (66.67%)	0.76 (95% CI 0.48–1.21)
Rural	46/51 (90.19%)	Ref.	44/53 (84.90%)	0.92 (95% CI 0.79–1.07)	14/16 (87.50%)	0.97 (95% CI 0.79–1.19)	18/25 (72.00%)	0.80 (95% CI 0.62–1.04)
PR^2^ (95% CI)	0.97 (95% CI 0.87–1.09)	–	1.15 (95% CI 1.01–1.32)	-	1.14 (95% CI 0.95–1.38)	–	0.93 (95% CI 0.55–1.56)	–

	**GROUP 2 (2019–2022)** **n** **=** **2,669**							
	<1989 *n* = 645		1989–1994 *n* = 832		1995–1996 *n* = 269		>=1997 *n* = 923	
Stratum	No (%)	PR^1^	No (%)	PR^1^	No (%)	PR^1^	No (%)	PR^1^
Total	581/645 (90.07%)	Ref.	773/832 (92.90%)	1.03 (95% CI 1.00–1.06)	223/269 (82.89%)	0.92 (95% CI 0.87–0.98)	799/923 (86.56%)	0.96 (95% CI 0.93–1.00)
Urban	350/386 (90.67%)	Ref.	396/424 (93.39%)	1.03 (95% CI 0.99–1.07)	85/96 (88.54%)	0.98 (95% CI 0.90–1.06)	222/263 (84.41%)	0.93 (95% CI 0.88–0.99)
Rural	231/259 (89.18%)	Ref.	377/408 (92.40%)	1.04 (95% CI 0.98–1.09)	138/173 (79.76%)	0.89 (95% CI 0.82–0.97)	577/660 (87.42%)	0.98 (95% CI 0.93–1.03)
PR^2^ (95% CI)	1.02 (95% CI 0.96–1.07)	–	1.01 (95% CI 0.97–1.05)	–	1.11 (95% CI 1.00–1.23)	–	0.97 (95% CI 0.91–1.02)	–

PR^1^, prevalence ratio relative to the group born before 1989, not eligible for anti-Rubella vaccine.

PR^2^, prevalence ratio in women from urban areas relative to women from rural areas, within the same eligibility subgroup.

Ref., reference level.

NA, not applicable (reference group had zero events).

Because this is a cross-sectional study, associations are expressed as prevalence ratios (PR) with 95% confidence intervals (CI), rather than risk ratios, following recommended practice for prevalence data (14). PR and 95% CI were calculated directly from the tabulated counts using the standard log-linear approximation.

### 3.3 Mapping of anti-Rubella IgG antibodies prevalence in dolj county between 2019 and 2022

Geographic distribution in Dolj County showed heterogeneous IgG seroprevalence (Table 6; Figure 3). Several localities reached 100% (Argetoaia, Breasta, Cârcea, Cetate, Daneți, and Poiana Mare), while lower seroprevalence was observed in specific towns (e.g., Șimnicu de Sus 57.14%, Ișalnița 64.29%). The IgG mapping suggests localized clusters of lower immunity that may warrant targeted public health attention, as well as a cluster in the center of the county around Craiova City with prevalences of 80%–90% and even more in some towns that extends in the north-east corner around the city of Filiași. The clustering can also reflect higher access to laboratory testing, as most labs in the county are located in Craiova and Filiasi.

**Table 6:** Seroprevalence of the IgG anti-Rubella antibodies in pregnant women – spread in dolj county, Romania 2019–2022.

Town	Seroprevalence of anti-Rubella IgG antibodies	Town	Seroprevalence of anti-Rubella IgG antibodies
Argetoaia	100%	Filiași	87.10%
Breasta	100%	Malu Mare	86.96%
Cârcea	100%	Mârșani	85.00%
Cetate	100%	Ciupercenii noi	84.21%
Daneți	100%	Bechet	83.33%
Poiana Mare	100%	Bucovăț	81.82%
Podari	97.62%	Caraula	81.82%
Calopăr	95.00%	Giurgița	81.82%
Valea Stanciului	92.86%	Plenița	81.82%
Brădești	92.86%	Moțăței	80.00%
Castranova	91.67%	Segarcea	80.00%
Coțofenii din față	91.67%	Teasc	80.00%
Amărăștii de jos	90.91%	Unirea	77.27%
Calafat	90.00%	Gruia	73.33%
Desa	90.00%	Bratovoiești	72.73%
Țuglui	90.00%	Goicea	72.73%
Craiova	89.56%	Băilești	72.50%
Pielești	88.89%	Bistreț	70.59%
Caracal	87.50%	Braloștița	70.00%
Coșoveni	87.50%	Ghindeni	66.67%
Dăbuleni	87.50%	Ișalnița	64.29%
Sadova	87.50%	Șimnicu de sus	57.14%

**Figure 3:**
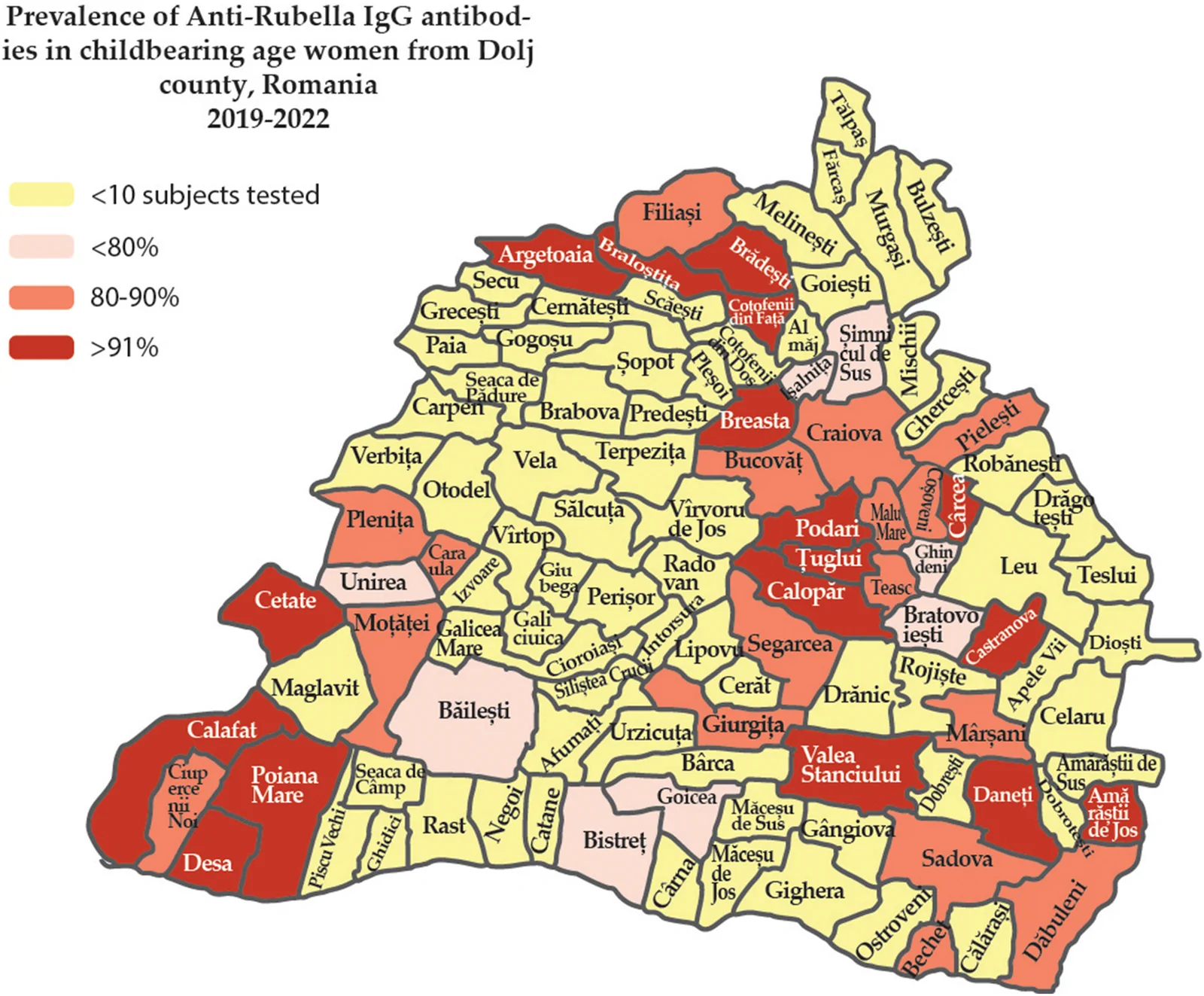
Igg anti-Rubella seroprevalence in pregnant women – spread in dolj county, Romania 2019-2022.

## 4 Discussion

This is the largest study performed in the South-West Romania regarding the seroprevalence of anti-Rubella antibodies in pregnant women, which came to complete the studies performed in other regions of Romania.

We observed a decrease in the mean age of pregnant women that addressed for testing anti-Rubella antibodies, which can reflect a decrease in the mean age of pregnant women in general or better access to healthcare for young pregnant women.

The increase of more than 7-fold in the number of pregnant women tested for anti-Rubella antibodies from 2013 to 2016 to 2019–2022 can be explained by the increase in medical information of patients and progress of laboratory technology associated with a reduction of testing costs, which become affordable for the average pregnant women (15, 16).

Because the national immunization program for rubella was introduced in Romania in 2003 (8), we expect a higher proportion of tested women under 20 years of age to be IgG-positive.

### 4.1 History of Rubella in Romania

In Romania, the first rubella cases were reported even since 1949 (17). In years 2002–2003, in Romania was recorded a large rubella outbreak (17), with over 115,000 reported cases, leading to an incidence rate of 531/100,000 inhabitants and 150 cases of suspected CRS, but only seven were confirmed. The epidemic affected mostly children, with an incidence rate of 2564/100,000 for the 5–9 years age group and 2446/100,000 in the 10–14 years age group.

As response measures, in the absence of an RCV, was implemented an outbreak response plan that included surveillance for anti-rubella antibodies in the affected children of pregnant women who either had rubella disease or had a history of contact with a confirmed rubella case.

This outbreak contributed to the acceleration of RCVs into the national vaccination programme.

Between 2011 and 2012 another rubella outbreak occurred in Romania, with reported incidence rates of 20.6/100,000 inhabitants in 2011 and 97.5/100,000 inhabitants in 2012 (18). During the same period, 137 cases of CRS were reported. Most IgM positive cases were in the 15–19-year-old age group, and 97.7% of cases had no history of vaccination.

### 4.2 Anti-Rubella IgM antibodies seroprevalence

Globally, according to the CDC, the number of reported rubella cases has declined from 94,277 in 2012 to 10,194 in 2020 (19). The CRS showed an increased incidence from 302 reported cases in 2012 to 603 reported cases in 2020. This apparent increase in incidence was actually a reporting bias, as new countries with large populations (Bangladesh, India, Indonesia, and Pakistan) were included in the WHO surveillance network (19).

In February 2020, 27 rubella cases were reported in six countries (Ireland, Germany, Italy, Poland, Portugal, and Norway), of which 18 were reported in Poland (20).

In our study, the prevalence of anti-rubella IgM antibodies was 0.54% between 2013 and 2016 (2 cases) and 0.50% (14 cases) between 2019 and 2022 (Table 3). A similar seroprevalence (0.531%) was reported in a rubella outbreak in Romania in 2003 (17). In a study performed during the 2010–2011 rubella outbreak in Romania, 88 (28.02%) of 314 pregnant women tested positive for IgM (a marked difference from the low, inter-epidemic seroprevalence found in the present study, consistent with the much higher pre-test probability of true infection during an active community outbreak, as opposed to the low-prevalence, endemic setting of our cohorts, where a larger relative proportion of IgM-positive results are expected to be false positives; see below) (18). A study performed in the same period reported a peak prevalence of 10.525% among children under 14 years of age from urban areas in Northern Romania (21). Between 2008 and 2010 in the Banat region (Western Romania), a seroprevalence of 0.479% was reported among women of childbearing age, compared with 0.273% between 2015 and 2018 (22). The results of our study confirmed this descending trend in IgM seroprevalence from 0.54% between 2013 and 2016 and 0.50% between 2019 and 2022. The ECDC website reported only four rubella cases in Romania during 2019–2020 and no reported cases since 2021 (20).

When interpreting the results of the IgM antibody test, we must consider the possibility of a false-positive result due to laboratory error. Even if modern methods for the detection of antibodies have high specificity, if many women are screened, there is a real possibility that some of the results are false positives (23). A study performed in the United States on pregnant women concluded that up to 11% of cases positive for IgM antibodies were actually negative (24). Also, is demonstrated cross reactivity between IgM anti-rubella antibodies and the rheumatoid factors that is present in blood in many auto-immune diseases (23).

A false-positive IgM result was documented in samples collected from women infected with viruses, such as parvovirus B19, or those that were positive for rheumatoid factor (18, 23).

### 4.3 Anti-Rubella IgG antibodies seroprevalence

The seroprevalence of anti-Rubella IgG antibodies of 88.28% during 2013–2016 and 88.91% during 2019–2022 was rather low compared to the WHO target of 95% seroprevalence rate (19). Globally, seroprevalence rates as low as 53% have been reported (10). In Europe, a meta-analysis found 16 studies reporting a seronegativity rate > 10% (<90% seropositivity) (25). The lowest reported seroprevalence was 81.2% in pregnant women in Greece in 2006, and the highest was 98.6% in women of childbearing age in the Czech Republic (26). The WHO sets a goal of eradication of rubella in Europe by 2030 (27).

Comparing the seroprevalences reported in our study with those reported in other regions of Romania, we observed that in Western Romania, higher seroprevalences were reported between 2008 and 2010 (94.1%) and 2015–2018 (91.5%) in women of childbearing age (22).

We did not observe a significant change in the overall seroprevalence between the two time periods (88.28% vs. 88.91%; *p* = 0.721) (Table 4). If we consider the age group, we observed a non-significant increase in seroprevalence in young women aged < 20 years, from 78.57% between 2013 and 2016 to 85.11% between 2019 and 2022 (*p* = 0.362). This numerically higher (though not statistically significant) seroprevalence can be tentatively explained if we consider the eligibility for vaccination because women that had 15–20 years between 2013 and 2016 were born between 1993 and 2000, which was confirmed by studies in Western Romania (28). Those born between 1993 and 1994 were eligible for the RCV vaccine, those born between 1995 and 1996 were not eligible for either vaccine, and those born between 1997 and 2000 were eligible for the second dose of the MMR vaccine (Table 1). In contrast, pregnant women aged 15–20 years between 2019 and 2022 were born between 1999 and 2004 and were eligible for at least one dose of MMR vaccine (Table 1), which explains the higher seroprevalence. In this age group, the (non-significant) difference was numerically larger in urban areas (66.67% vs. 77.94%, *p* = 0.529) than in rural areas (81.82% vs. 87.38%, *p* = 0.462) (Table 4). Another age group in which we saw an increase in seroprevalence (from 82.50% to 92.42%) was 31–35 years. These women were not eligible for an RCV vaccine; thus, the increase in seroprevalence can be attributed to natural immunization. This could be due to two rubella outbreaks that occurred in Romania in 2003 and 2011–2012 (17, 18). A study performed in 1989 (29) showed a seroprevalence of 75.3% in this age group, whereas another study from Western Romania reported a seroprevalence in women aged 26–34–93.6% between 2008 and 2010 and 91.8% between 2015 and 2018. We can conclude that the seroprevalence is on an ascending trend in Romania from 1989 to 2022. This is despite governmental data showing a decrease in vaccination coverage from 93.2% in 2010 to 89.6% in the first dose and to 80.9% in the second dose in 2018 (28). This decrease is due to a continuous increase in MMR vaccine hesitation due to unconfirmed reports of adverse effects (28). Vaccine hesitation has been reported in more than 90% of the countries worldwide (13). Globally, the WHO reports in 2021 an MMR vaccination coverage of 71% for the first dose and 81% for the second dose, at the lowest level since 2008 (30). In 2021, 71% of children received both doses of the measles vaccine, and approximately 81% of the world's children received one dose of the measles vaccine by their first birthday, the lowest since 2008. Furthermore, the COVID-19 pandemic disrupted routine immunization programs and contributed to a decline in vaccination coverage (World Health Organization 2023b).

In the USA, among children aged 19–35 months, vaccination coverage decreased from 91.6% in 2011 to 91.5% in 2017 (13). In Europe, in the UK, vaccination coverage in children at 24 months dropped by 79.9% in 2003–2004, comparative with >90% in the 1990s (31).

In Romania, the last report of MMR vaccination coverage (February 2023) showed a percentage of 78% in children aged 18 months, which was much lower than in 2011, when it was 93.2% (32).

Some explanations for vaccine hesitation could be parents' refusal due to unconfirmed reports about vaccine-related adverse effects or doubts of vaccine benefits. In 1998, a study reported a possible connection between the MMR vaccine and autism in children (33), which was the seed for a controversy concerning the adverse effects of the MMR vaccine, which eventually spread into websites and social media that contributed to parental misinformation. Numerous studies rejected this link, and eventually, the article was retracted (34). Another study from 2012 suggested a link between the MMR vaccine and aseptic meningitis (35).

Despite this retraction, this unfounded claim remains a point of contention among parents. It is imperative for physicians to proactively address these concerns by highlighting the proven benefits of immunization, the potential risks of infections, and the established safety record of vaccines. While it is crucial to acknowledge that no medical intervention, including vaccines, has potential side effects, most reactions to MMR vaccines are mild, such as fever, lymphadenopathy, rash, or parotitis. More severe adverse reactions, including febrile seizures, anaphylaxis, thrombocytopenic purpura, and encephalitis, are exceedingly rare (36). Notably, MMR vaccines using the Urabe and Leningrad-Zagreb mumps strains have been linked to aseptic meningitis (35).

We observed a higher seroprevalence in the urban areas than in rural areas in Group 1 (89.64% vs. 86.21%, *p* = 0.480, not statistically significant) (Table 4), a pattern also reported by Mocanu et al. between 2015 and 2018 (22), whereas in Group 2, urban seroprevalence remained slightly higher than rural seroprevalence (89.82% vs. 88.20%, *p* = 0.935, not statistically significant), consistent with the pattern observed in Group 1. In a study performed during the 2003 rubella outbreak, the number of cases was higher in urban areas owing to increased population density (21). Another explanation for this could be the easier access to vaccination in urban areas. In rural areas, there is increased vaccination refusal due to lower income and socioeconomic status, lower health education, and a higher number of children per family (37). Comparing the two time periods, rural seroprevalence changed from 86.21% to 88.20% (PR = 1.02, 95% CI 0.96–1.09; *p* = 0.480), a numerically small difference that was not statistically significant; a similarly small, non-significant change was seen in urban areas (89.64% to 89.82%; PR = 1.00, 95% CI 0.95–1.05; *p* = 0.935) (Table 4). This is consistent with a generally stable level of protective immunity in both settings across the study period, rather than a genuine improvement, and should be interpreted with the general improvement of the healthcare system and vaccination access in mind only as a plausible, unconfirmed explanation. In Romania reports from 2023, MMR vaccine coverage is 76.5% in rural areas, almost three percent lower than that in urban areas (79.5%) (32).

We observed that the IgG seroprevalence increased with age of the pregnant women from 85.11% in women aged < 20 years to 92.42% in women aged 31–35 years. This can be correlated with the reported decrease in vaccination coverage from 2010 to 2018 (6, 20). The same trend was confirmed in England and Belgium (26). In other countries (Bulgaria and Slovenia Croatia), age was unrelated to seroprevalence (14, 26), while Hungary and Lithuania even reported a decrease in seroprevalence with age (26).

The most important finding of this study is the reduced IgG seroprevalence in women born during the 1995–1996 vaccination gap and in those born in or after 1997, despite theoretical eligibility for rubella-containing vaccines. These results suggest that vaccination eligibility does not equate to effective or durable immunity at the population level. The convergence of IgG seroprevalence between rural and urban areas over time likely reflects improved healthcare access and screening coverage; however, overall immunity remains insufficient to prevent CRS. These findings support the need for targeted catch-up vaccination strategies, particularly for women of reproductive age in identified susceptible cohorts.

### 4.4 Mapping of anti-Rubella IgG antibodies prevalence in dolj county between 2019 and 2022

The distribution of anti-rubella IgG antibody seroprevalence in towns from Dolj County showed relatively high rates in all analyzed towns, as explained by the vaccination program with the second dose that was administered in schools. In some towns such as Argetoaia, Breasta, Cârcea, Cetate, Daneți, and Poiana Mare, the 100% seroprevalence rates indicate that all pregnant women from these towns were exposed to the disease or vaccine. Conversely the seroprevalence rates are low in towns like Șiminicu de sus (57.14%) and Ișalnița (64.29%), that can suggest a lower rate of vaccination.

However, these data should be interpreted in context, as the seroprevalence rate can be influenced by numerous factors, which can vary between towns. For example, seroprevalence can differ between towns with different population structures according to the age group. If the mean age is low in a certain town, the seroprevalence can be higher owing to more recent vaccination. In towns near large hospitals, access to healthcare is easier, which can result from a higher vaccination rate or easier access to testing, due to the short distance and easier access to public transportation, suggesting a lot of movement between towns. This can explain the higher seroprevalence rates observed in Calafat (90.00%), Craiova (89.56%) and Filiași (87.10%). In this way, the virus can spread easily in peripheral towns, affecting the seroprevalence rates. Additionally, modern immunological tests, such as anti-rubella antibodies, are generally not available in small laboratories in the countryside.

### 4.5 Limitations

There are potential confounding factors that can distort the observed seroprevalence, as we did not collect, for example, the vaccination status of our subjects, as the anti-Rubella vaccine was included in the national immunization program in 2004 (38). We only collected the demographic factors of the patients, but other factors, such as education level and socioeconomic status, were shown to influence the seroprevalence rate (37). Consequently, the majority of the pregnant women tested were vaccinated. In constructing the prevalence map, we used only the data from 2019 to 2022, because the hospital electronic records were implemented only after 2016, and we could not find the town information for the subjects presented at the hospital between 2013 and 2016. The two study periods differed considerably in sample size (367–368 in Group 1 vs. 2,669–2,779 in Group 2 for IgG/IgM, corresponding to margins of error of approximately ±2.9% and ±1.1%, respectively, for an expected prevalence around 90%). Although this increased the precision of estimates in the later period, it may have affected the power and stability of comparisons involving the earlier cohort, and some between-group differences that did not reach statistical significance (e.g., the increase in IgG seroprevalence among women <20 years of age, or the small decrease in IgM seroprevalence) may partly reflect the wider confidence intervals of the smaller Group 1 sample rather than a true absence of difference. This should be kept in mind when interpreting comparisons between the two periods, particularly for the least frequent subgroups.

This study was designed to estimate rubella antibody seroprevalence in pregnant women and was not designed, and did not collect data, to assess clinical outcomes of IgM-positive pregnancies. We did not collect individual clinical follow-up data, maternal symptoms, treatment, or newborn outcomes (including congenital rubella syndrome status, gestational age at birth, birth weight, or stillbirth) for IgM-positive women, and the hospital electronic database used for this study does not link maternal serology records to newborn clinical outcomes. Consequently, we are unable to report on maternal or neonatal clinical outcomes associated with IgM positivity in this cohort; this represents an important area for dedicated prospective follow-up studies, distinct from the seroprevalence-survey design used here.

As discussed above, the use of two different immunoassay platforms (ECLIA and CMIA) across the two study periods is a further limitation, since published multicentric evaluations indicate that quantitative and, near the cut-off, qualitative rubella IgG results can differ between commercial platforms despite common calibration to the WHO International Standard (14, 38). We cannot exclude a contribution of this inter-assay variability to the between-period comparisons reported here.

## 5 Conclusions

This study provides updated evidence on rubella immunity among pregnant women in South-Western Romania. Acute rubella infection was rare across both study periods, while overall anti-rubella IgG seroprevalence remained stable but consistently below the 95% threshold associated with WHO elimination targets. Although rural–urban differences diminished over time, population-level immunity remains insufficient to prevent congenital rubella syndrome.

The identification of lower IgG seroprevalence in specific birth cohorts, particularly women born during the 1995–1996 vaccination gap and those born in the MMR-eligible era, highlights persistent immunity gaps despite long-standing vaccination programs. These findings suggest that vaccination eligibility alone does not guarantee adequate protection in women of reproductive age.

Strengthening rubella prevention in South-Western Romania requires targeted catch-up vaccination strategies, enhanced counseling by family physicians, and sustained surveillance of susceptible cohorts. Addressing these immunity gaps is essential for achieving rubella and congenital rubella syndrome elimination goals.

## Data Availability

The original contributions presented in the study are included in the article/Supplementary Material, further inquiries can be directed to the corresponding authors.

## References

[1] Mankertz A Chen MH Goldberg TL Hübschen JM Pfaff F Ulrich RG. ICTV Virus taxonomy profile: matonaviridae 2022. J Gen Virol. (2022) 103. 10.1099/JGV.0.001817 36748520 PMC12643105

[2] World Health Organization Rubella (German Measles) Factsheet. Available online at: https://www.who.int/news-room/fact-sheets/detail/rubella (Accessed May 1, 2025).

[3] European Centre for Disease Prevention and Control (ECDC) Surveillance Atlas of Infectious Diseases. Available online at: https://atlas.ecdc.europa.eu/public/index.aspx?Dataset=27&HealthTopic=45 (Accessed May 1, 2025).

[4] Krawiec C Hinson JW. Rubeola (measles). Pathology Pathogenesis Human Viral Disease. San Diego: Academic Press (2023). p. 397–410. 10.1016/b978-012195160-3/50030-3

[5] Shukla S Maraqa NF. Congenital Rubella. In: StatPearls. Treasure Island, FL: StatPearls Publishing (2023). 29939656

[6] World Health Organization, *Global Measles and Rubella Strategic Plan: 2012-2020*. Geneva: WHO Press (2012).

[7] World Health Organization Rubella Vaccination Coverage Available online: Available online at: https://immunizationdata.who.int/pages/coverage/RCV.html?CODE=Global&YEAR= (Accessed July 28, 2023).

[8] European Centre for Disease Prevention and Control (ECDC) Vaccination Coverage for the First Dose of Rubella-Containing Vaccine by Country, EU/EEA, 2017. Available online at: https://www.ecdc.europa.eu/en/publications-data/vaccination-coverage-first-dose-rubella-containing-vaccine-country-eueea-2017 (Accessed July 28, 2023).

[9] Institutul Național de Statistică. Proiectarea Populației României, Pe Regiuni de Dezvoltare Și Județe, La Orizontul Anului 2070 | Institutul Național de Statistică. Available online at: https://insse.ro/cms/ro/content/proiectarea-popula%C8%9Biei-rom%C3%A2niei-pe-regiuni-de-dezvoltare-%C8%99i-jude%C8%9Be-la-orizontul-anului-2070 (Accessed August 11, 2026).

[10] Onakewhor JU Chiwuzie J. Seroprevalence survey of Rubella infection in pregnancy at the university of Benin teaching hospital, Benin city, Nigeria. Niger J Clin Pract*.* 2011 14:140–5. 10.4103/1119-3077.84002 21860127

[11] Bouthry E Furione M Huzly D Ogee-Nwankwo A Hao LJ Adebayo A. Assessing immunity to Rubella virus: a plea for standardization of IgG (immuno)Assays. J Clin Microbiol. (2016) 54:1720–5. 10.1128/JCM.00383-16 27147722 PMC4922105

[12] Tamhane AR Westfall AO Burkholder GA Cutter GR. Prevalence odds ratio versus prevalence ratio: choice Comes with consequences. Stat Med. (2016) 35:5730–5. 10.1002/SIM.7059 27460748 PMC5135596

[13] The Lancet Child Adolescent Health. Vaccine hesitancy: a generation at risk. Lancet Child Adolesc. Health (2019) 3(5):281. 10.1016/S2352-4642(19)30092-6 30981382

[14] Vilibic-Cavlek T Ljubin-Sternak S Ban M Kolaric B Sviben M Mlinaric-Galinovic G. Seroprevalence of TORCH infections in women of childbearing age in Croatia. J Matern Fetal Neonatal Med. (2011) 24:280–3. 10.3109/14767058.2010.485233 20476874

[15] World Health Organization. Romania: Health System Review 2016. Health Systems in Transition, vol. 18 (2016). Available online at: https://iris.who.int/items/f7bf6fab-1812-45a4-9f6e-164435f1025f (Accessed May 1, 2025).

[16] Purcărea VL Coculescu BI Coculescu EC. Improving the quality of health care in the Romanian public health system - a priority in the reform process. J Med Life. (2015) 8:166. PMC4392101 25866573

[17] Rafila A Marin M Pistol A Nicolaiciuc D Lupulescu E Uzicanin A. A large Rubella outbreak, Romania–2003. Euro Surveill. (2004) 9:7–9. 10.2807/ESM.09.04.00457-EN 15192257

[18] Lazar M Abernathy E Chen M Icenogle J Janta D Stanescu A. Epidemiological and molecular investigation of a Rubella outbreak. Romania. 2011 to 2012. Eurosurveillance. (2016) 21(38):30345. 10.2807/1560-7917.ES.2016.21.38.30345 PMC5073198 27684329

[19] Zimmerman LA Knapp JK Antoni S Grant GB Reef SE. Progress toward Rubella and congenital Rubella syndrome control and elimination - worldwide, 2012–2020. MMWR Morb Mortal Wkly Rep. (2022) 71:196–201, 10.15585/MMWR.MM7106A2 35143468 PMC8830626

[20] Monthly Measles and Rubella Monitoring Report - April 2020 (2020).

[21] Costan OC Manole A. Epidemiologic research on Rubella incidence in Various demographic structures, in a north-eastern area of Romania. Rev Med Chir Soc Med Nat Iasi. (2004) 108:880–5. 16004235

[22] Mocanu AG Gorun F Ciohat I Navolan D Malita D Vilibic-Cavlek T. Simultaneous seroprevalence to toxoplasma gondii, cytomegalovirus and Rubella virus in childbearing women from western Romania. Medicina (Kaunas). (2021) 57:927. 10.3390/MEDICINA57090927 34577850 PMC8469601

[23] Isaac BM Zucker JR Giancotti FR Abernathy E Icenogle J Rakeman JL. Rubella surveillance and diagnostic testing among a low-prevalence population New York city 2012–2013. Clin Vaccine Immunol*.* (2017) 24:e00102–17. 10.1128/CVI.00102-17 28701468 PMC5585696

[24] Averhoff F Zucker J Vellozzi C Redd S Woodfill C Waterman S. Adequacy of surveillance to detect endemic Rubella transmission in the United States. Clin Infect Dis. (2006) 43 Suppl 3:S151–7. 10.1086/505948 16998775

[25] Pandolfi E Gesualdo F Rizzo C Bella A Agricola E Mastroiacovo P. Global seroprevalence of Rubella among pregnant and childbearing age women: a meta-analysis. Eur J Public Health. (2017) 27:530–7. 10.1093/EURPUB/CKW259 28204465

[26] Nardone A Tischer A Andrews N Backhouse J Theeten H Gatcheva N. Comparison of Rubella seroepidemiology in 17 countries: progress towards international disease control targets. Bull World Health Organ. (2008) 86:118–25. 10.2471/BLT.07.042010 18297166 PMC2647372

[27] World Health Organization. *Measles and Rubella Strategic Framework 2021–2030*. Geneva: WHO Press (2020).

[28] Gorun F Malita D Ciohat I Vilibic-Cavlek T Feier H Tabain I. Prevalence of Rubella antibodies among Fertile women in the west of Romania, 18 years after the implementation of immunization. Vaccines (Basel)*.* (2021) 9:104. 10.3390/VACCINES9020104 33572943 PMC7912329

[29] Dumitrescu R Mateescu M Gaicu N Comanescu D. Evaluation of the anti-Rubella immunity levels on a lot of 5,000 Sera from women at procreative age, tested by HAI, in Romania. Arch Roum Pathol Exp Microbiol. (1989) 48:253–63. 2519635

[30] World Health Organization. Measles. Available online at: https://www.who.int/news-room/fact-sheets/detail/measles (Accessed August 26, 2023).

[31] National Health Service (NHS) *Childhood Vaccination Coverage Statistics*, England 2010-2011. Leeds: NHS Digital (2022).

[32] Institul național de Sănătate Publică Romania *Analiza Rezultatelor Estimarii Acoperirii Vaccinale La Varsta de 18 Luni Februarie* 2023. 2023.

[33] Wakefield AJ Murch SH Anthony A Linnell J Casson DM Malik M. Retracted: ileal-lymphoid-nodular hyperplasia, non-specific colitis, and pervasive developmental disorder in children. Lancet. (1998) 351:637–41. 10.1016/S0140-6736(97)11096-0 9500320

[34] Godlee F Smith J Marcovitch H. Wakefield's article linking MMR vaccine and autism was fraudulent. Br Med J. (2011) 342:64–6. 10.1136/BMJ.C7452 21209060

[35] Demicheli V Rivetti A Debalini MG Di Pietrantonj C. Vaccines for measles, mumps and Rubella in children. Cochrane Database Syst Rev*.* (2012) (2):CD004407. 10.1002/14651858.CD004407.pub3 PMC6458016 22336803

[36] Bouthry E Picone O Hamdi G Grangeot-Keros L Ayoubi JM Vauloup-Fellous C. Rubella and pregnancy: diagnosis, management and outcomes. Prenat Diagn. (2014) 34:1246–53. 10.1002/PD.4467 25066688

[37] Tabacchi G Costantino C Napoli G Marchese V Cracchiolo M Casuccio A. on behalf of the ESCULAPIO working group, Amicizia D Bechini A, Determinants of European Parents' Decision on the vaccination of their children against measles, mumps and Rubella: a systematic review and meta-analysis. Hum Vaccin Immunother*.* (2016) 12:1–15:1909. 10.1080/21645515.2016.1151990 27163657 PMC4964810

[38] National Institute of Public Health. Center for monitoring and control of transmissible diseases. Analysis of Evolution of Transmissible Diseases That Are Monitored. Report for the Year 2015. Bucharest, (2016).

