# Supplementary material for: Anti-Rubella antibodies seroprevalence among pregnant women in South-Western Romania
Source: Front Med (Lausanne). 2026 Sep 24;13:1906798. doi: 10.3389/fmed.2026.1906798 (PMC13645689; doi:10.3389/fmed.2026.1906798)
Supplement: Supplementary file 1 [file Table1.docx]

Table S1. Anti-rubella IgM positivity in pregnant women in 2013–2016 (Group 1) versus 2019–2022 (Group 2), overall and stratified by area of residence and age group. Differences between periods were assessed using chi-square/Fisher’s exact tests as appropriate; p < 0.05 was considered statistically significant.

| Variable | Group 1 2013-2016  (n=368) | Group 2 2019-2022  (n=2,779) | p value |
| --- | --- | --- | --- |
| Total prevalence | 2/368 (0.54%) | 14/2,779 (0.50%) | 0.920 |
| Area of residence |  |  |  |
| Rural | 1/145 (0.69%) | 9/1,564 (0.58%) | 0.863 |
| Urban | 1/223 (0.45%) | 5/1,215 (0.41%) | 0.937 |
| Age group |  |  |  |
| <20 years | 0/28 (0%) | 1/316 (0.32%) | 0.766 |
| 21-25 years | 2/79 (2.53%) | 6/709 (0.85%) | 0.153 |
| 26-30 years | 0/130 (0%) | 4/797 (0.5%) | 0.418 |
| 31-35 years | 0/80 (0%) | 3/630 (0.48%) | 0.536 |
| >35 years | 0/51 (0%) | 0/327 (0%) | 0.999 |
| Area of residence and age group |  |  |  |
| Rural, <20 years | 0/22 (0%) | 1/239 (0.42%) | 0.761 |
| Rural, 21-25 years | 1/45 (2.17%) | 5/491 (1.02%) | 0.462 |
| Rural, 26-30 years | 0/44 (0%) | 2/417 (0.48%) | 0.645 |
| Rural, 31-35 years | 0/21 (0%) | 1/279/0.36%) | 0.783 |
| Rural, >35 years | 0/12 (0%) | 0/138 (0%) | 0.999 |
|  |  |  |  |
| Urban, <20 years | 0/6 (0%) | 0/77 (0%) | 0.999 |
| Urban, 21-25 years | 1/33 (3.03%) | 1/218 (0.46%) | 0.999 |
| Urban, 26-30 years | 0/86 (0%) | 2/380 (0.53%) | 0.580 |
| Urban, 31-35 years | 0/59 (0%) | 2/351 (0.57%) | 0.561 |
| Urban, >35 years | 0/39 (0%) | 0/189 (0%) | 0.999 |

* Statistical significance (p<0.05)

IgM positivity remained <1% across most strata; comparisons were underpowered due to low event counts.

Table S2. Anti-rubella IgM positivity by vaccination-eligibility birth cohort (<1989, 1989–1994, 1995–1996, ≥1997), overall and stratified by urban/rural residence, in 2013–2016 (Group 1) and 2019–2022 (Group 2). RR1 indicates risk ratio versus the <1989 reference cohort; RR2 compares urban versus rural within each cohort.

| **GROUP 1 (2013-2016)**  **n=368** | | | | | | | |  |
| --- | --- | --- | --- | --- | --- | --- | --- | --- |
|  | <1989  n=184 | | 1989-1994  n=124 | | 1995-1996  n=25 | | >=1997  n=35 | |
|  | No (%) | RR^1^  (p) | No (%) | RR^1^  (p) | No (%) | RR^1^  (p) | No (%) | RR^1^  (p) |
| Total | 0/184  (0%) | Ref. | 2/124 (1.61%) | 6.57*10^7^  (0.996) | 0/25  (0%) | 1  (1.000) | 0/35  (0%) | 1  (1.000) |
| Urban | 0/51  (0%) | Ref. | 1/53 (1.88%) | 1.11*10^8^ | 0/15  (0%) | 1  (1.000) | 0/26  (0%) | 1  (1.000) |
| Rural | 0/133  (0%) | Ref. | 1/71 (1.41%) | 3.72*10^7^ | 0/10  (0%) | 1  (1.000) | 0/9  (0%) | 1  (1.000) |
| RR^2^  (p) | NA |  |  | 1.02  (0.988) |  | NA |  | NA |
|  |  |  |  |  |  |  |  |  |
| **GROUP 2 (2019-2022)**  **n=2,779** | | | | | | | | |
|  | <1989  n=666 | | 1989-1994  n=855 | | 1995-1996  n=280 | | >=1997  n=978 | |
|  | No (%) | RR^1^  (p) | No (%) | RR^1^  (p) | No (%) | RR^1^  (p) | No (%) | RR^1^  (p) |
| Total | 1/666 (0.15%) | Ref. | 5/855 (0.58%) | 3.72  (0.240) | 2/280 (0.71%) | 4.69  (0.208) | 6/978 (0.61%) | 4.00 (0.199) |
| Urban | 0/396  (0 %) | Ref. | 4/438 (0.91%) | 1.31*10^7^  (0.993) | 0/104  (0%) | 1  (1.000) | 1/277 (0.36%) | 1.08*10^10^  (0.994) |
| Rural | 1/270 (0.37%) | Ref. | 1/417 (0.24%) | 0.74 (0.837) | 2/176 (1.13%) | 3.04 (0.365) | 5/701 (0.71%) | 1.90 (0.557) |
| RR^2^  (p) | NA | - |  | 3.14  (0.293) | - | NA | - | 0.52  (0.543) |
|  |  |  |  |  |  |  |  |  |

RR^1^ – risk ratio relative to cohort born <1989 (reference).

RR^2^ – risk ratio for urban versus rural residence within the same birth cohort

Ref. – Reference level for multinomial logistic regression

NA – Not Applicable
